# Biventricular Increases in Mitochondrial Fission Mediator (MiD51) and Proglycolytic Pyruvate Kinase (PKM2) Isoform in Experimental Group 2 Pulmonary Hypertension-Novel Mitochondrial Abnormalities

**DOI:** 10.3389/fcvm.2018.00195

**Published:** 2019-01-25

**Authors:** Ping Yu Xiong, Lian Tian, Kimberly J. Dunham-Snary, Kuang-Hueih Chen, Jeffrey D. Mewburn, Monica Neuber-Hess, Ashley Martin, Asish Dasgupta, Francois Potus, Stephen L. Archer

**Affiliations:** ^1^Department of Medicine, Queen's University, Kingston, ON, Canada; ^2^Department of Biomedical and Molecular Sciences, Queen's University, Kingston, ON, Canada; ^3^Queen's Cardiopulmonary Unit, Department of Medicine, Queen's University, Kingston, ON, Canada

**Keywords:** aortic stenosis, PKM2 pyruvate kinase M2, mitochondrial fission, MiD51, supra-coronary aortic banding (SAB), group 2 PH, pulmonary hypertension, mitochondrial pyruvate carrier

## Abstract

**Introduction:** Group 2 pulmonary hypertension (PH), defined as a mean pulmonary arterial pressure ≥25 mmHg with elevated pulmonary capillary wedge pressure >15 mmHg, has no approved therapy and patients often die from right ventricular failure (RVF). Alterations in mitochondrial metabolism, notably impaired glucose oxidation, and increased mitochondrial fission, contribute to right ventricle (RV) dysfunction in PH. We hypothesized that the impairment of RV and left ventricular (LV) function in group 2 PH results in part from a proglycolytic isoform switch from pyruvate kinase muscle (PKM) isoform 1 to 2 and from increased mitochondrial fission, due either to upregulation of expression of dynamin-related protein 1 (Drp1) or its binding partners, mitochondrial dynamics protein of 49 or 51 kDa (MiD49 or 51).

**Methods and Results:** Group 2 PH was induced by supra-coronary aortic banding (SAB) in 5-week old male Sprague Dawley rats. Four weeks post SAB, echocardiography showed marked reduction of tricuspid annular plane systolic excursion (2.9 ± 0.1 vs. 4.0 ± 0.1 mm) and pulmonary artery acceleration time (24.3 ± 0.9 vs. 35.4 ± 1.8 ms) in SAB vs. sham rats. Nine weeks post SAB, left and right heart catheterization showed significant biventricular increases in end systolic and diastolic pressure in SAB vs. sham rats (LV: 226 ± 15 vs. 103 ± 5 mmHg, 34 ± 5 vs. 7 ± 1 mmHg; RV: 40 ± 4 vs. 22 ± 1 mmHg, and 4.7 ± 1.5 vs. 0.9 ± 0.5 mmHg, respectively). Picrosirius red staining showed marked biventricular fibrosis in SAB rats. There was increased muscularization of small pulmonary arteries in SAB rats. Confocal microscopy showed biventricular mitochondrial depolarization and fragmentation in SAB vs. sham cardiomyocytes. Transmission electron microscopy confirmed a marked biventricular reduction in mitochondria size in SAB hearts. Immunoblot showed marked biventricular increase in PKM2/PKM1 and MiD51 expression. Mitofusin 2 and mitochondrial pyruvate carrier 1 were increased in SAB LVs.

**Conclusions:** SAB caused group 2 PH. Impaired RV function and RV fibrosis were associated with increases in mitochondrial fission and expression of MiD51 and PKM2. While these changes would be expected to promote increased production of reactive oxygen species and a glycolytic shift in metabolism, further study is required to determine the functional consequences of these newly described mitochondrial abnormalities.

## Introduction

Group 2 pulmonary hypertension (PH), also known as PH due to left heart disease (PH-LHD), is defined by the combination of elevated resting mean pulmonary arterial pressure (mPAP) (≥25 mmHg) and elevated left heart filling pressures (pulmonary capillary wedge pressure (PCWP) >15 mmHg and left ventricle end diastolic pressure (LVEDP) >18 mmHg (1). Left ventricular myocardial diseases and left-sided valvular diseases are the commonest causes of Group 2 PH (1).

Currently, there are no approved PH-targeted therapies for group 2 disease, even though it is the most prevalent form of PH (2). Existing treatments focus on symptom relief and correction of the underlying LHD. However, group 2 PH typically cannot be completely reversed by treating LHD. For example, 78% of mitral stenosis (MS) patients develop PH (3). After successful mitral valve replacement surgery, PH persists in approximately half of patients (4). This sustained PH may relate to the persistence of adverse pulmonary vascular remodeling, RV fibrosis and/or RV contractile dysfunction. With the goal of better understanding the pathophysiology and underlying molecular mechanisms of group 2 PH, we evaluated a promising mitochondrial metabolic pathway that has been identified in studies of Group 1 PH (5–8). We used a well-validated rat model of group 2 PH created by supra-coronary aortic banding (SAB) (9–11).

In right ventricular hypertrophy (RVH) associated with group 1 PH, the most prominent pathway alterations at the transcriptomic level involve mitochondria and are predicted to result in mitochondrial dysfunction (8). Consistent with this, there are two pathophysiological relevant mitochondrial abnormalities commonly seen in the RV in group 1 PH. First, there is a metabolic shift toward uncoupled glycolysis and away from glucose oxidation, the Warburg phenomenon, reviewed in Piao et al. (12). Second, there is an increase in mitochondrial fission (7). In group 1 PH, activation of pyruvate dehydrogenase kinase (PDK) inhibits pyruvate dehydrogenase (PDH) contributing to RVF. Moreover, increased expression/activity of dynamin-related protein-1 (DRP1)-mediated fission elevates the production of mitochondria-derived reactive oxygen species, which contributes to RV dysfunction (7). While restoring glucose oxidation or inhibiting fission are beneficial in group 1 PH models (7, 13), it is unknown whether similar mechanisms occur in group 2 PH.

It is also noteworthy that proglycolytic mechanisms occur in Group 1PH, at least in the pulmonary vasculature, in addition to PDK activation (14, 15). Notably there is dysregulation of pyruvate kinase (PK), the final and rate-limiting step in glycolysis. PK transfers phosphate from phosphoenolpyruvate to ADP, producing pyruvate and ATP, reviewed in Archer (16). Acquired changes in the ratio of pyruvate kinase muscle (PKM) splice variant 2 vs. 1 (specifically and increase in the expressed PKM2/PKM1 ratio) occurs in endothelial cells and fibroblasts of group 1 PH patients and contributes to increases in glycolysis and cell proliferation while reducing apoptosis rates (14, 15).

Drp1 is also known to mediate mitochondrial fission in the LV. Inhibiting Drp1 improves LV function and prevents ventricular remodeling in the transverse aortic constriction mouse model (17). The Drp1 inhibitor mdivi-1 reduces cardiomyocyte apoptosis and promotes angiogenesis (17). Inhibiting mitochondrial fission preserves LV function in a global ischemia model and, in a rodent cardiac arrest model, mdivi-1 enhances the success of resuscitation (6, 18). However, Drp1 activity relates to many parameters in addition to expression level, notably its interaction with binding partners on the outer mitochondrial membrane, including mitochondrial fission factor (MFF), Fission 1 (Fis1), and mitochondrial dynamics proteins of 49 and 51 kDa (MiD49 and MiD51) (19). The role of the changes in expression of PK splice variants and MiDs in the heart is unstudied in group 2 PH.

In this study we evaluated two hypotheses: First, that the impairment of RV function in group 2 PH is associated in part with a biventricular proglycolytic isoform switch from PKM1 to PKM2 predominance. Second, that RVH in group 2 PH is associated with increased mitochondrial fission, associated with upregulation of expression of Drp1 and/or its binding partners, MiD49 and MiD51.

## Materials and Methods

The experimental protocol has been approved by Queen's University Animal Care Committee and the University Research and Ethic Board. All animals are raised in the Queen's Animal Care Facility.

### The SAB Model Experimental Design

Five-week-old male Sprague Dawley rats (Charles River, Montreal, QC) were raised in the Queen's Animal Care Facility. After a week of acclimatization, SAB or sham surgery was performed on the rats to produce SAB and sham groups. Each experimental cohort was composed of *n* = 10 rats (4 sham and 6 SAB) and underwent surgery in the same week. A total of 2 cohorts were performed for this study (total *n* = 20). Echocardiography was performed 4 weeks post-surgery. Terminal catheterization and tissue collection were done 9 weeks post-surgery (Supplemental Figure 1).

### SAB Surgery

A 2 cm skin incision was made between the second and third ribs and, subsequently, a 1.5 cm incision of the intercostal muscle layer. The ribs were retracted and the ascending aorta visualized (Supplemental Figures 2A–C). A size small (3 × 3mm) Weck® Horizon™ titanium clip (Catalog #523735, Teleflex, Markham, ON) was placed around the ascending aorta using an applicator. The clip immediately constricts the aorta to ~50% of the original diameter (Supplemental Figures 2D,E). Following aortic constriction, the ribs were approximated with interrupted 4-0 Vicryl™ (polyglactin) sutures (Supplemental Figure 2F). The ventilator was paused for 3 s and air aspirated using a 23G chest-tube in order to re-inflate the lungs. The muscle layers and the skin were opposed with 4–0 Vicryl™ (polyglactin) sutures and surgical clips were applied over the skin suture (Supplemental Figures 2G,H). After surgery, rats were returned to their cages, positioned on their sides and observed until they were awake and in stable condition. Standard post-operative care was provided to minimize pain and risk of wound infection.

### Echocardiography

Estimation of pulmonary arterial pressure (PAP) was done using Doppler ultrasound. Serial 2-dimensional, M-mode and pulsed-wave Doppler ultrasound recordings were performed under anesthesia (inhaled, isoflurane, 1.6–2.0%, mixed with humidified medical air delivered via a cone inhaler). Cardiovascular imaging was performed using a Vevo® 2100 (FUJIFILM VisualSonics Inc, Toronto, ON) phased-array, color-Doppler ultrasound system with a 37.5 MHz transducer and frame rates up to 1,000/s. Ultrasound studies were performed weekly beginning 1 week post-surgery to measure tricuspid annular plane systolic excursion (TAPSE), right ventricular wall thickness, and pulmonary artery acceleration time (PAAT). Mean PAP is inversely related to PAAT and TAPSE is directly related to RV function.

### Catheterization and Euthanasia

Rats were anesthetized with isoflurane (1.6–2.0%). SBP, LVESP, and LVEDP were measured in closed-chest rats with a 1.9-F rat pressure-volume catheter (Scisense Inc. London, ON), which was introduced via the right common carotid artery. RVESP and RVEDP were measured from RV pressure-volume loops created by introduction of the same catheter into the LV via the right common carotid artery, which was introduced via the right jugular vein. Finally, the rat was euthanized by exsanguination while deeply anesthetized.

### Tissue Collection

Tissues were harvested immediately following hemodynamic measurements. The heart was washed and dissected in phosphate buffered saline (PBS) solution at 0°C and the RV was separated from the left ventricle plus septum (LV+S) and weighed. The RV and LV+S were cut into 3–4 small pieces, frozen in liquid nitrogen, and stored at −80°C. Samples of both ventricles were also fixed with formalin for histology. Picrosirius red staining was used to measure fibrosis. The lung was processed similarly. The left lower lobe was inflated and fixed with formalin and later stained with hematoxylin and eosin (H&E).

### Western Blot

Tissues (LV, RV, and lung) were flash frozen and ground into fine powder using a mortar and pestle. Tissue powder lysates were prepared in cell lysis buffer (Cell Signaling Technologies, Beverly MA, USA). For immunoblot analysis, tissue lysates (40–80 μg) were analyzed on 4–12% NuPAGE gels (Life technologies, Carlsbad, CA, USA). The proteins were electrotransferred to a polyvinylidene difluoride (PVDF) membrane (Life technologies, Carlsbad, CA, USA) and detection of specific proteins was carried out with the antibodies indicated below, using the ECL-Plus Western Blotting Detection System (GE Healthcare, Piscataway, NJ, USA). Total Drp1 (611112) antibody was purchased from BD transduction Laboratories (San Jose, Ca, USA). Antibodies were obtained from: MiD49 (16413-1-AP), MiD51 (20164-1-AP), PKM1 (15821-1-AP), and PKM2 (15822-1-AP) from Proteintech (Tucson, AZ, USA), Mfn2 (ab56889), pPDH (ab92696), and PDH (ab110334) from Abcam (Cambridge MA, USA),. Vinculin (V9131) and Mitochondrial pyruvate carrier 1 antibodies (MPC1) from Sigma-Aldrich (St. Louis, MO, USA) (SAB4502158). Mitochondrial pyruvate carrier 2 antibodies (MPC2) (MABS1914) were obtained from Millipore (Temecula, CA, USA).

### Assessment of Mitochondrial Membrane Potential in Cardiac Tissues

Mitochondrial membrane potential was qualitatively assessed in LV and RV muscle section using tetramethylrhodamine methyl ester (TMRM; Cat # T668, Life Technologies; Carlsbad, CA, USA). After hemodynamic study, the rat was sacrificed, and the isolated LV and RV tissue were immediately incubated in Krebs' solution containing TMRM (250 nM) at 37°C for 30–45 min and NucBlue® Live Ready Probes® Reagent (2 drops/mL), following the manufacturer's protocol (Cat # R37605, Life Technologies; Carlsbad, CA, USA). The tissue was imaged using a Leica SP8 confocal, laser-scanning microscope (Leica Microsystems; Wetzlar, Germany) with a 1.40 NA, 63x oil immersion objective (~ 4/frames/minute for 5–10 min; the microscopist was blinded to the treatment group). TMRM intensity was measured using ImageJ software (National Institutes of Health; Bethesda, MD, USA). The microscopist was blinded to the treatment group. More details can be found in previously published study (7).

### Transmission Electron Microscopy (TEM)

TEM of the LV and RV were performed on tissues fixed in osmium tetroxide using an FEI Tecnai Osiris Transmission Electron Microscope, as previously described (7).

### Pyruvate Dehydrogenase (PDH) Enzyme Activity Dipstick Assay

PDH enzyme activity was measured in the LV and RV of SAB vs. Sham rats using frozen tissue powder lysates in cell lysis buffer (Cell Signaling Technologies, Beverly MA, USA). The samples were then applied to PDH enzyme activity dipstick assay following the manufacturer's protocol (Cat# ab109882, Abcam, Cambridge MA, USA), as previously described (20).

### Statistical Analysis

Values are expressed as mean ± standard error of mean (SEM). Statistical comparisons between sham vs. SAB rats were performed using unpaired, parametric, two-tailed Student's *t*-test. *P*-values ≤ 0.05, ≤ 0.01, ≤ 0.001, and ≤ 0.0001 are designated with ^*^, ^**^, ^***^, and ^****^, respectively. Statistical calculations were performed using GraphPad Prism 7 (GraphPad Software, Inc., La Jolla, CA, USA).

## Results

**Figure 1:**
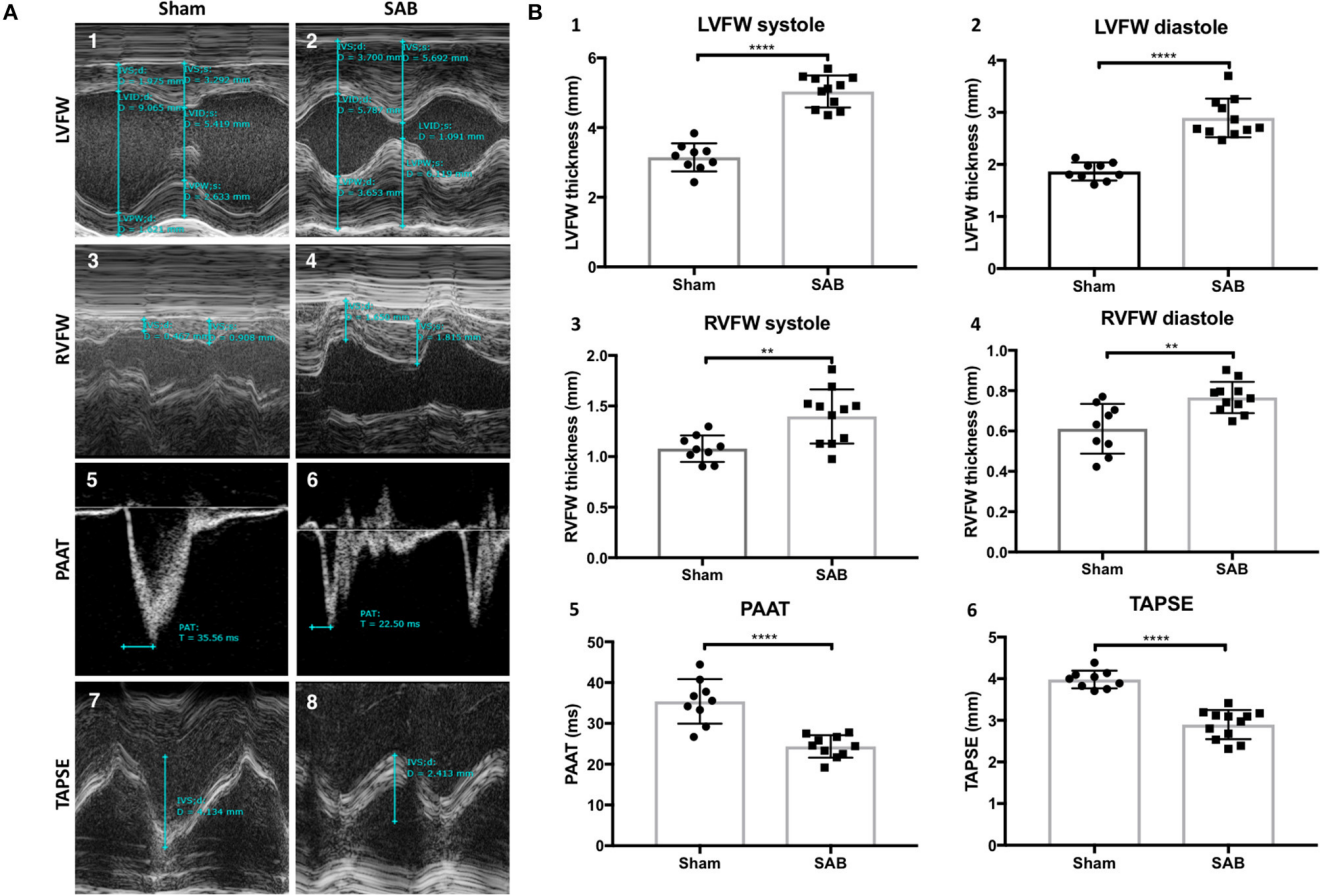
Echocardiographic measurements of sham vs. supra-coronary aortic banding (SAB) rats at 4 weeks post banding. **(A)** Representative echocardiographic images showing development of group 2 PH in SAB rats. (1–2) Left ventricular free wall thickness (LVFW), (3–4) right ventricular free wall thickness (RVFW), and (5–6) pulmonary artery acceleration time (PAAT), and (7–8) tricuspid annular plane systolic excursion (TAPSE) of sham vs. supra-coronary aortic banding (SAB) rats. **(B)** Summary statistics showing significant increase of biventricular thickness and decrease of PAAT and TAPSE in SAB vs. sham. (1) LVFW thickness, (2) diastolic LVFW thickness, (3) RVFW thickness, (4) diastolic RVFW thickness, (5) PAAT, (6) TAPSE of sham vs. supra-coronary aortic banding rats.

SAB surgery induced moderate PH. Four weeks post-SAB, echocardiography showed a significant increase of LV free wall (LVFW) thickness, systolic (5.04 ± 0.14 vs. 4.15 ± 0.13 mm, *p* < 0.0001) and diastolic (2.89 ± 0.11 vs. 1.86 ± 0.06 mm, *p* < 0.0001). There was also an increase in both systolic and diastolic RV free wall (RVFW) thickness (1.40 ± 0.08 vs. 1.08 ± 0.04 mm, *p* = 0.0044, and 0.77 ± 0.02 vs. 0.61 ± 0.04 mm, *p* = 0.0030, respectively). PAAT was reduced in SAB vs. sham (24.3 ± 0.9 vs. 35.4 ± 1.8 ms, *p* < 0.0001), as was the TAPSE (2.9 ± 0.1 vs. 4.0 ± 0.1 mm, *p* < 0.0001) (Figures 1A,B). Respiratory rate was significantly increased in the SAB vs. sham rats (70 ± 4 vs. 53 ± 3 breath/minute, *p* = 0.0062), but heart rate was not significantly different between the two groups (Supplemental Figure 3).

**Figure 2:**
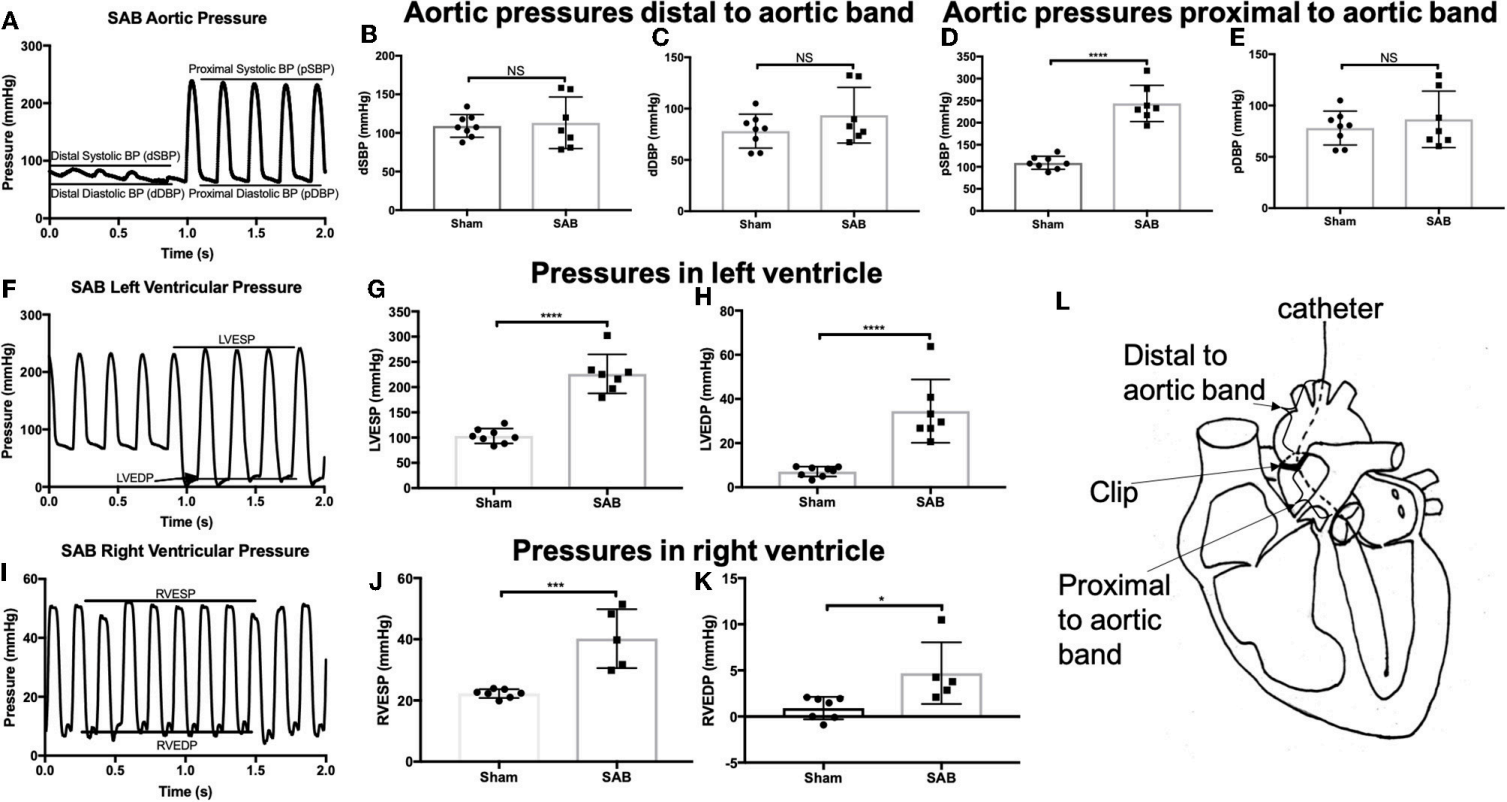
Hemodynamic studies of sham vs. SAB rats. Left carotid catheterization showing **(A)** hemodynamic measurement of **(B)** systolic, and **(C)** diastolic blood pressure distal to the band (dSBP and dDBP) and **(D)** systolic and **(E)** diastolic blood pressure proximal to the band (pSBP and pDBP). Left heart catheterization showing **(F)** hemodynamic measurement of **(G)** left ventricular end systolic pressure (LVESP) and **(H)** left ventricular end diastolic pressure (LVEDP). Right heart catheterization showing **(I)** hemodynamic measurement of **(J)** right ventricular end systolic pressure (RVESP) and **(K)** right ventricular end diastolic pressure (RVEDP). **(L)** Schematic drawing showing the anatomical definition of distal and proximal to aortic band.

Left heart catheterization showed no significant difference in the systolic and diastolic blood pressure distal to the band (dSBP and dDBP, Figures 2A–C,L). As expected, systolic blood pressure proximal to the band (pSBP) was significantly increased in SAB vs. sham rats (244 ± 16 vs. 109 ± 5 mmHg, *p* < 0.0001, Figure 2D) without alteration of diastolic blood pressure (pDBP, Figure 2E). LV end systolic pressure (LVESP) and end diastolic pressure (LVEDP) were increased in SAB vs. control rats (226 ± 15 vs. 103 ± 5 mmHg, *p* < 0.0001) and (34 ± 5 vs. 7 ± 1 mmHg, *p* < 0.0001), respectively (Figures 2F-H). Right heart catheterization showed significant increase of RV end systolic and diastolic pressure (40 ± 4 vs. 22 ± 1 mmHg, *p* = 0.0006, and 4.7 ± 1.5 vs. 0.9 ± 0.5 mmHg, *p* = 0.02, respectively) in SAB vs. sham rats (Figures 2I–K).

**Figure 3:**
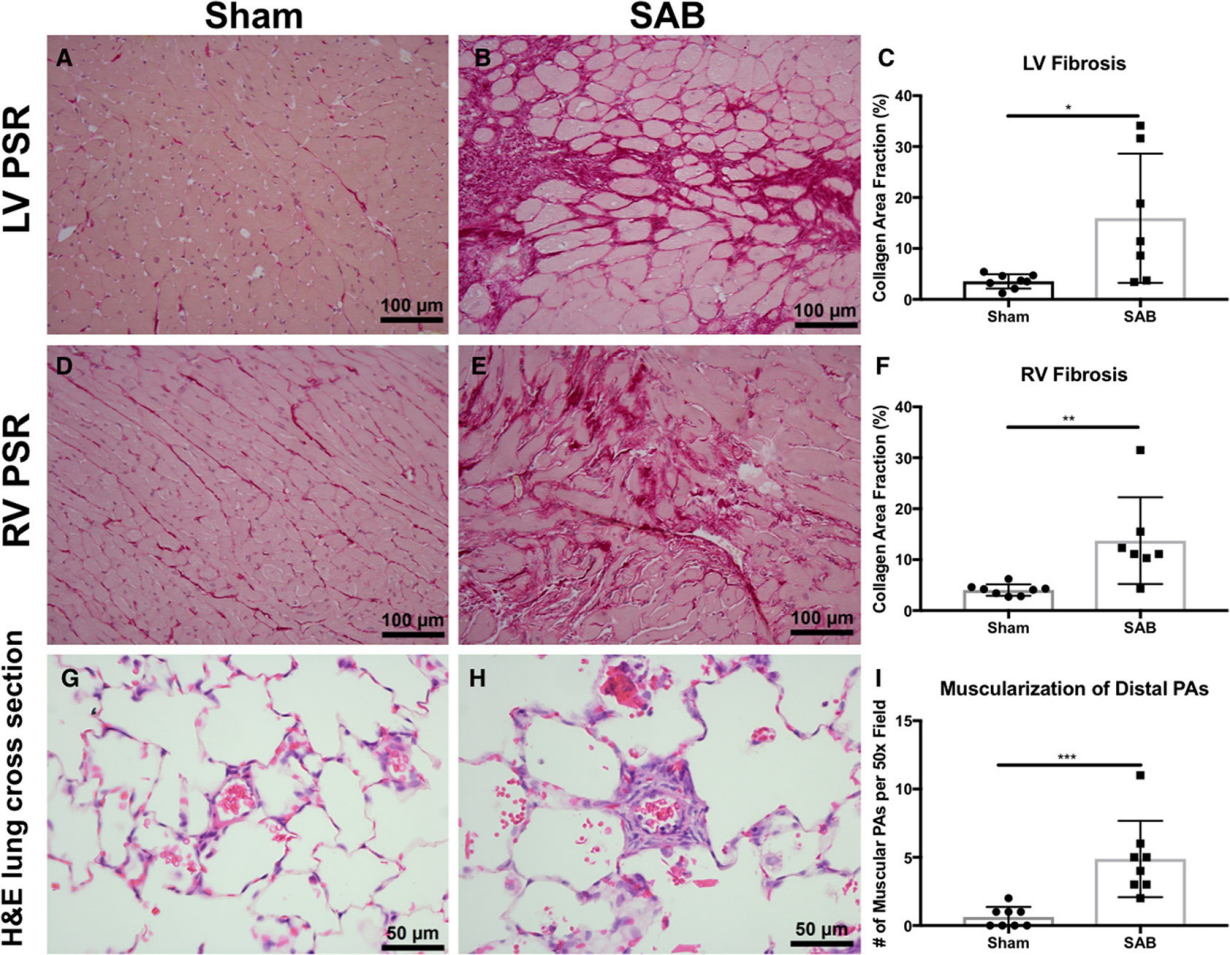
Histology and confocal microscopy images of sham vs. supra-coronary aortic banding (SAB) rats. Picrosirius red (PSR) staining of left ventricle (LV), and right ventricle (RV) of **(A,D)** sham vs. **(B,E)** SAB rats showing **(C,F)** increased myocardial fibrosis in SAB rat. Hematoxylin and eosin **(H,E)** stain of lung cross section of **(G)** sham vs. **(H)** SAB rats showing **(I)** increased number of muscular pulmonary arteries (PAs) per 50x field. Each dot represents tissue from a unique rat.

**Figure 4:**
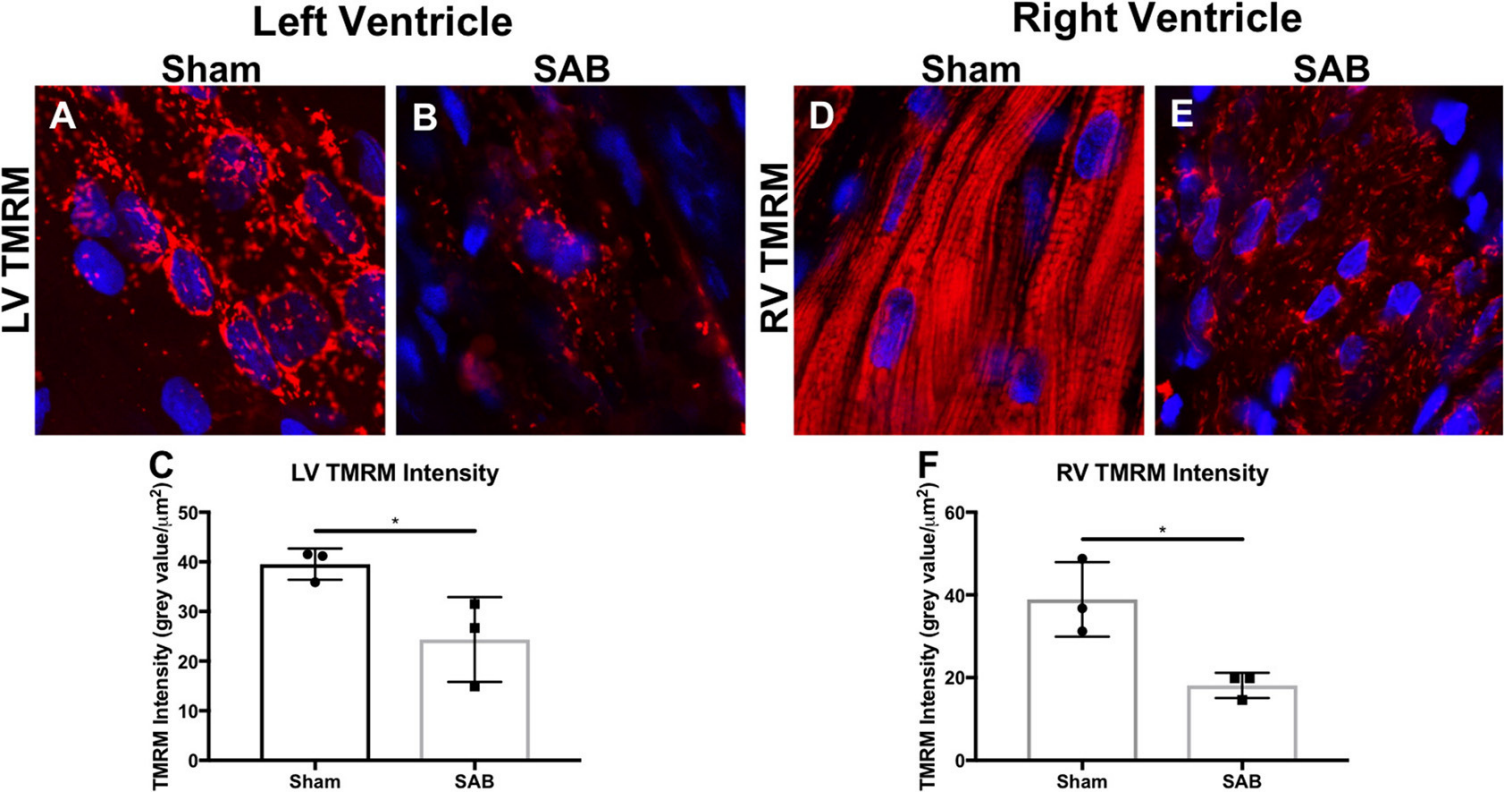
Representative confocal microscopy image showing left ventricular (LV) and right ventricular (RV) mitochondrial morphology and membrane potential of **(A,D)** sham rats vs. **(B,E)** supra-coronary aortic banding rats. The mitochondria (red) are labeled with tetramethylrhodamin, methyl ester (TMRM) and the nuclei (blue) are labeled with DAPI. **(C)** LV and **(F)** RV TMRM intensity. Each dot represents a heart from a unique rat.

**Figure 5:**
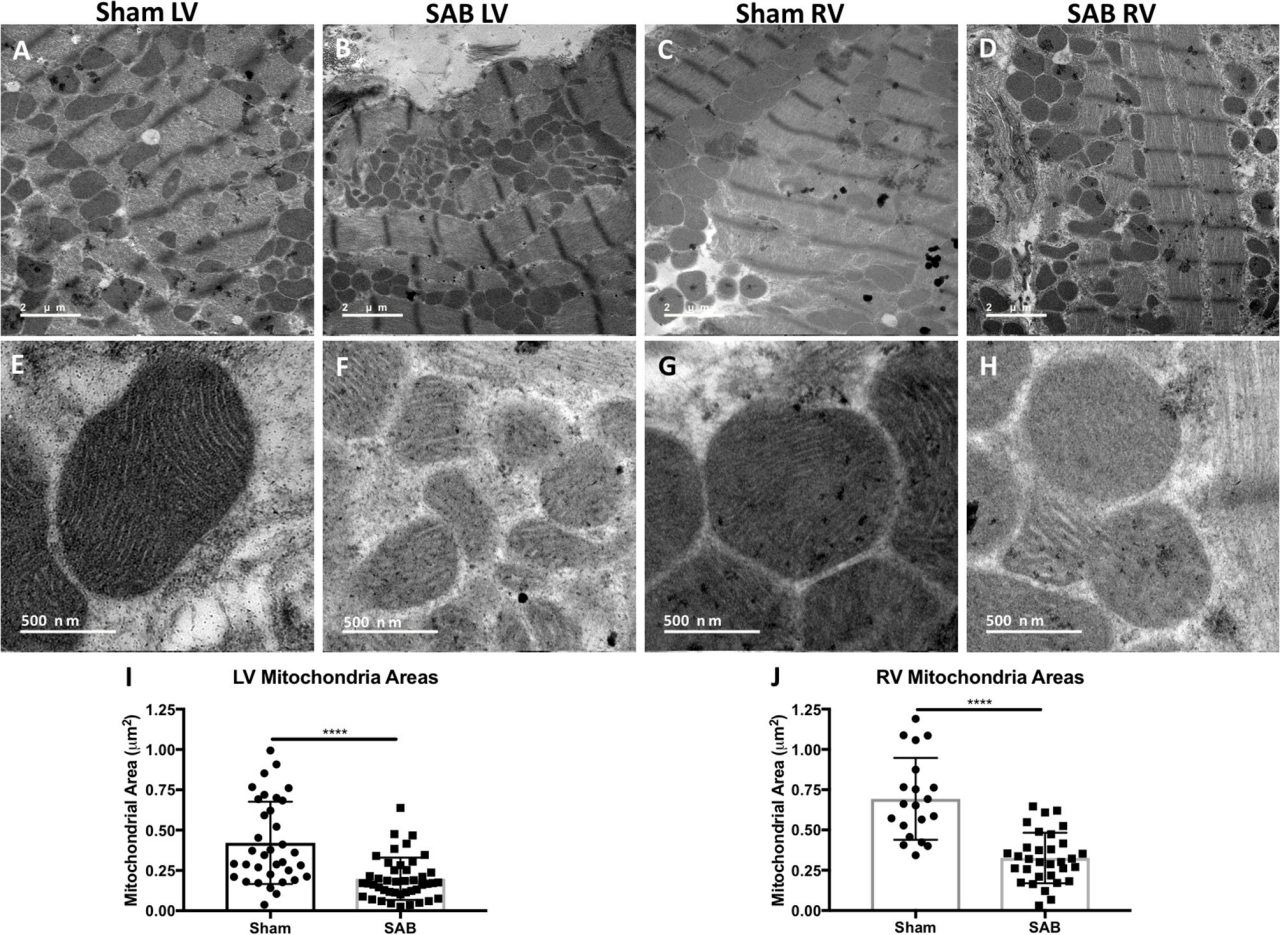
Representative transmission electron microscopy (TEM) images showing reduced average mitochondria area in the left ventricle (LV) and right ventricle (RV) at 2 different magnifications. **(A)** Low magnification of sham LV, **(B)** supra-coronary aortic banding (SAB) LV, **(C)** sham RV, and **(D)** SAB LV. High magnification showing mitochondrial ultra-sturcture in **(E)** sham LV, **(F)** SAB LV, **(G)** sham RV, and **(H)** SAB RV. **(I)** Average LV mitochondrial area, and **(J)** average RV mitochondrial area. Each dot represents a single mitochondrion.

There was increased fibrosis in both the LV and RV of SAB vs. sham rats (Figures 3A–F). Quantitative histology showed increased muscularization of small pulmonary arterioles (Figures 3G–I). TMRM staining showed mitochondria fragmentation (Figures 4A,B,D,E) and reduced TMRM intensity (Figures 4C–F) in the cardiomyocytes of SAB vs. sham rats. TEM showed that SAB caused mitochondrial fission with decreased average area/mitochondria in both LV (0.42 ± 0.04 vs. 0.20 ± 0.02 μm^2^, *p* < 0.0001) and RV (0.69 ± 0.06 vs. 0.33 ± 0.03 μm^2^ *p* < 0.0001) (Figures 5I,J).

**Figure 6:**
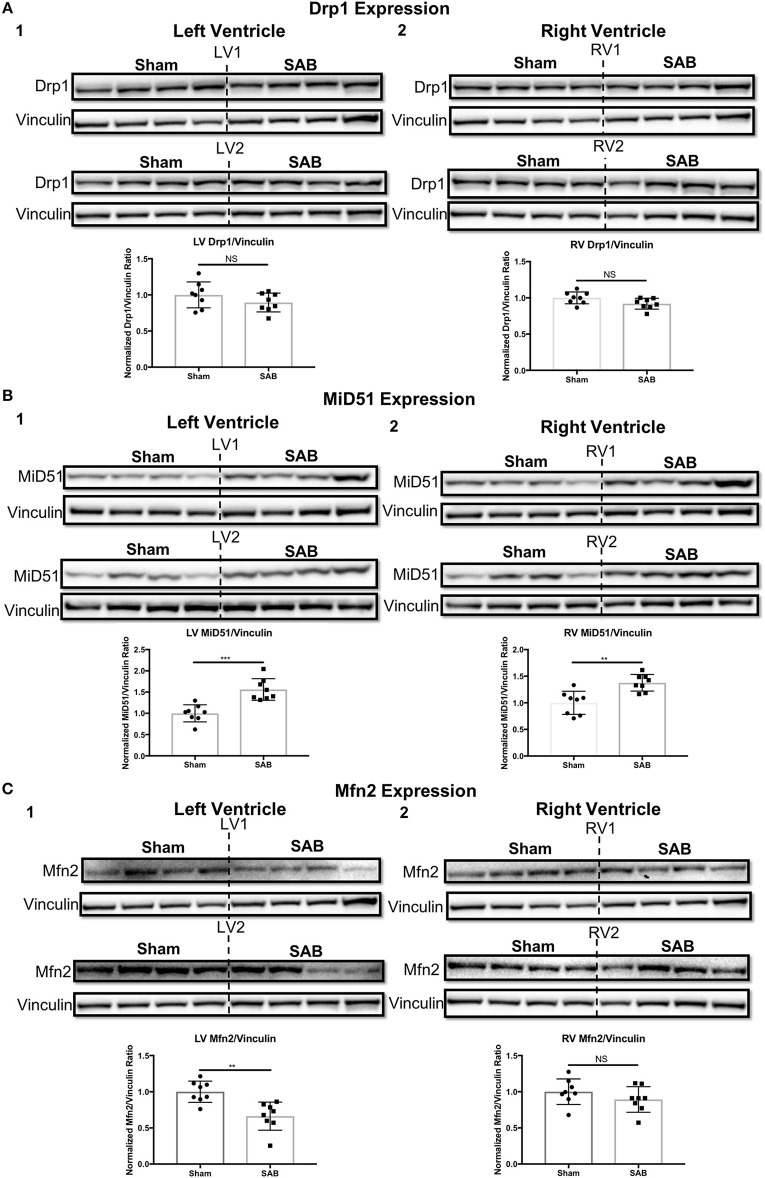
Expression levels of mitochondrial fission/fusion mediators in sham vs. supra-coronary aortic banding (SAB) rats. **(A)** Western blot showing no change in the expression level of total dynamin-related protein 1 (Drp1) in the (1) left ventricle (LV) and (2) right ventricle (RV) of supra-coronary aortic banding (SAB) rats vs. sham rats. **(B)** Western blot showing significant upregulation of mitochondrial dynamics of 51 kDa protein (MiD51) in the (1) LV and (2) RV of supra-coronary aortic banding (SAB) rats vs. sham rats. **(C)** Western blot showing significant downregulation of mitofusin-2 (Mfn2) in the (1) LV and (2) RV of supra-coronary aortic banding (SAB) rats vs. sham rats. Each band represents a unique animal. Two experimental cohorts were done; hence two groups are shown, labeled as 1 and 2. The same vinculin loading control is used for proteins probed on the same membrane.

**Figure 7:**
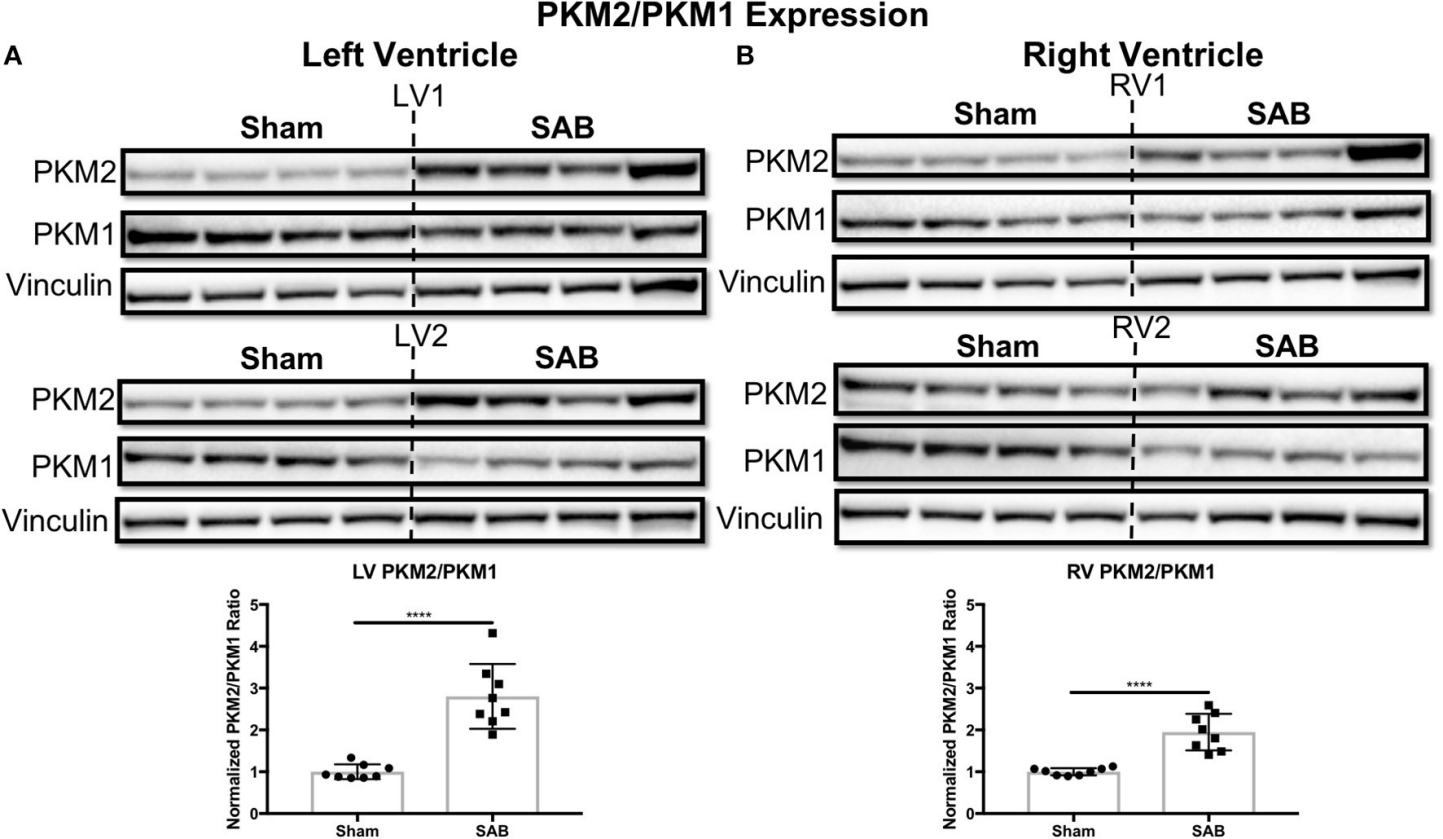
Pyruvate kinase muscle (PKM) isoform 2 to 1 ratio is increased in supra-coronary aortic banding (SAB) vs. sham rats. Western blot showing upregulation of PKM2 and downregulation of PKM1, thus resulting in significant increase of PKM2/PKM1 ratio in the **(A)** left ventricle (LV) and **(B)** right ventricle (RV) of supra-coronary aortic banding (SAB) rats vs. sham rats. Each band represents a unique animal. Two experimental cohorts were done; hence two groups are shown, labeled as 1 and 2. The same vinculin loading control is used for proteins probed on the same membrane.

**Figure 8:**
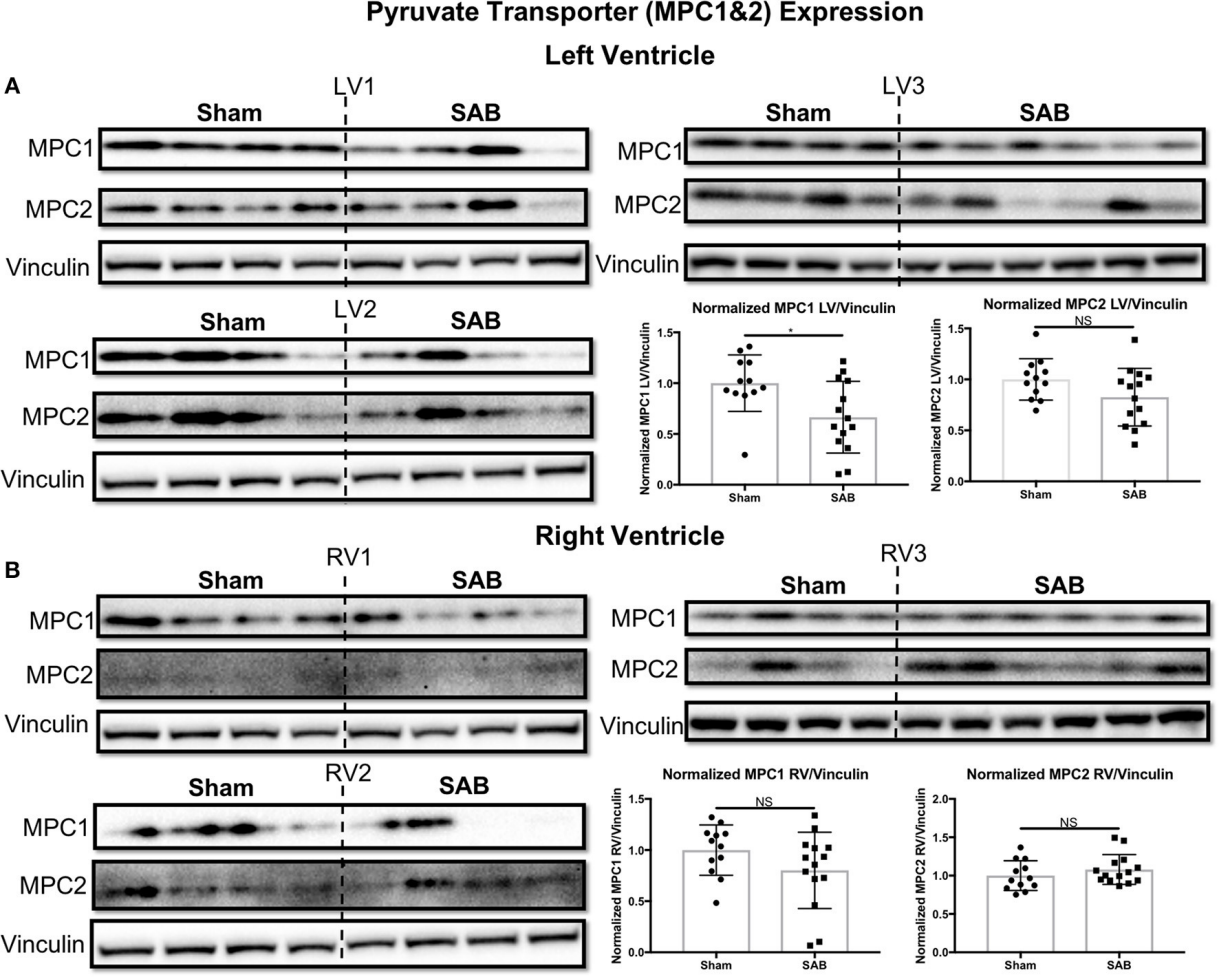
Mitochondrial pyruvate carrier 1 (MPC1) is upregulated in supra-coronary aortic banding (SAB) vs. sham rats. **(A)** Western blot showing significant upregulation of MPC1, but no change in MPC2 level in the left ventricle (LV) of SAB vs. sham rats. **(B)** Western blot showing no significant change of MPC 1&2 expression in the right ventricle (RV) of SAB vs. sham rats. Each band represents a unique animal. Three experimental cohorts are done; hence three groups are shown, labeled as 1–3. The same vinculin loading control is used for proteins probed on the same membrane.

Western blot showed no change in the expression of total Drp1 in the LV and RV of SAB vs. sham rats (Figure 6A). However, there was a significant, biventricular increase in the expression MiD51 (but not MiD49) in both the LV (*p* = 0.0002) and RV (*p* = 0.0014) of SAB vs. sham rats (Figure 6B and Supplemental Figure 4, respectively). Mfn2 expression was significantly decreased in the LV (*p* = 0.0016) (Figure 6C1) but was unchanged in the RV in SAB vs. sham rats (Figure 6C2). PKM2 was significantly upregulated and PKM1 was downregulated in both ventricles resulting in significant increase of PKM2 to PKM1 ratio in the LV (*p* < 0.0001) and RV (*p* < 0.0001) (Figure 7). MPC1 was decreased only in the LV (*p* = 0.0488) of SAB rats (Figures 8A,B). Neither the pPDH to PDH ratio (Supplemental Figure 5) nor the RV PDH enzyme activity (Supplemental Figure 6) was altered in SAB vs. sham rats.

## Discussion

This study has 3 major findings: (1) the SAB model reliably produces group 2 PH that is associated with RV fibrosis and dysfunction as well as adverse pulmonary vascular remodeling; (2) the group 2 PH is associated with a biventricular increase in the PKM2/PKM1 expression ratio in SAB rats, potentially priming the ventricles for a glycolytic shift in metabolism; (3) there is a biventricular increase in mitochondrial fission that is associated with an increase in MiD51 expression.

Most studies using the SAB rodent model focus on systemic hemodynamics and remodeling of the LV. In this study, we focuses on evaluating the SAB as a means of creating a robust preclincial model of group 2 PH. Methodologically our study differs from the few papers that also examine the effects of SAB on pulmonary hemodynamics and/or the RV, in that we use a surgical clip to constrict the aorta uniformly without circulatory interruption, as previously described (10, 21). In contrast, most groups studying group 2 PH have used a ligature to create the stenosis (9, 11, 22). In these studies, an 18-gauge blunted needle is placed parallel to the ascending aorta and a non-dissolvable suture tied snugly around the aorta and the needle (11). The needle is then removed leaving behind a fixed-size stenosis. In transverse aortic constriction (TAC) mice, a 26-gauge needle is typically used (22). In our experience, the suture method suffers from high intra-operative mortality and inconsistency in the degree of constriction/size of the stenosis. In contrast, our operations are successful with low mortality (~5% initial and ~10% at 30 days) and low variability (trans-clip pressure gradient 134.8 ± 15.5 mmHg). The clip method reduces operative mortality, decreases wound size, and increases reproducibility.

To our knowledge, this is the first evidence that chronic pressure overload alone can induce significant increase of biventricular PKM2/PKM1 ratio *in vivo*. PKM2 upregulation has been reported as a signature in the failing heart both in sunitinib-treated mice and human subjects (23). Shirai et al. report upregulation of PKM2 in macrophages from patients with atherosclerotic coronary artery disease compared to control subjects, suggesting PKM2 promotes a proinflammatory state (24). Two recent studies by Caruso et al. and Zhang et al. report increased PKM2/PKM1 ratio in the pulmonary endothelial and fibroblast cells from animal models and human subjects with group 1 PH, respectively (14, 15). In addition, there are numerus studies on the role of PKM2 in cancer (25) and, most recently, in inflammation (26). Despite these studies, the mechanism of pressure overload-induced PKM2/PKM1 upregulation remains elusive, because pressure overload not only increases the workload of the heart, but it also induces other changes, such as increased fibrosis (Figures 3C,F). Hence, upregulation of PKM2/PKM1 in SAB rats cannot all be explained by metabolic adaption in response to increased cardiac workload. Fibrotic response is usually a result of inflammation (27), and aortic banding induces inflammation (28). Emerging evidence suggests a correlation between increased PKM2 and proinflammatory state (24, 26). Thus, SAB-induced inflammation may be another mechanism that results in the upregulation of PKM2/PKM1. Future studies treating SAB rats with anti-inflammatory strategies, which has been shown to reduce cardiac fibrosis (29), may restore normal PKM2/PKM1 ratios.

Furthermore, an increased PKM2/PKM1 ratio in both ventricles is expected to result in a proglycolytic shift and causes simultaneous decreased pyruvate production and increased lactate production in the SAB vs. sham rats (16). This is expected to contribute to LV and RV dysfunction in this model. We do not expect nor observe an increase in pPDH (Supplemental Figure 5), since this post-translationally inactive form of PDH largely reflects increased pyruvate dehydrogenase kinase activation. Consistent with this, there are no significant changes in PDH enzyme activity based on the PDH dipstick assay (Supplemental Figure 6), suggesting that in the SAB model of group 2 PH the isoform switch from PKM1 to PKM2 is the predominant metabolic switch. Downregulation of MPC1 in the LV suggests that not only is there a shift to glycolysis but also that pyruvate transport into mitochondria may be impaired (Figure 8A).

Recently, Liang et al. report that PKM2 translocates to mitochondria under oxidative stress (30). Furthermore, Wu et al. show that PKM2 overexpression inhibits the expression of Drp1 and induces mitochondrial fusion in HeLa and HCT-116 cells (31). However, we observe that mitochondria within the cardiomyocytes of both ventricles are fragmented and depolarized (Figures 4, 5). The increased mitochondrial fission, evident from both confocal and TEM imaging in the LV and RV of SAB rats, is associated with increased MiD51. The mechanism for upregulation of MiD51 is not assessed in this study. However, knowing that PKM2 translocates to mitochondria (30) and affects Drp1 expression (31), it would be interesting to study the relationship between PKM2 and MiD51 in the future. In addition, a decrease in expression of the fusion mediator Mfn2 (Figure 6C) may also contribute to the observed mitochondrial fragmentation. Ryan et al. observed decreased expression of Mfn2 in lung samples from PAH patients and female PAH animal models (32). In this study, we observe significantly decreased expression of Mfn2 in the LV but not the RV.

### Limitations

Male Sprague-Dawley rats treated with SAB developed elevated LVEDP (34 ± 5 vs. 7 ± 1 mmHg), which meets the definition for group 2 PH (LVEDP > 18 mmHg). One limitation of this study is the lack of direct measurement of PAP. The use of a straight conductance catheter does not allow placement of the catheter in the PA. Thus, we can only measure RVESP, which is significantly elevated in SAB vs. sham rats (40 ± 4 vs. 22 ± 1 mmHg). RVESP is a close estimate of pulmonary arterial systolic blood pressure. However, we cannot measure PA diastolic blood pressure. Nonetheless, given the large rise in RVESP, significant increase of RVFW thickness, and shortening of PAAT on echocardiography we can assume the PAP is significantly increased in SAB vs. sham rats despite the lack of direct measurement.

Mitochondria respiration is not reported in this study because of large variation and poor reproducibility when we attempted to measure isolated mitochondria from heart tissue using the Seahorse analyzer.

Total Drp1 did not change in SAB vs. sham rats (Figure 6A). Another major limitation of this study is the lack phospho-Drp1-Ser616 (pDrp1-S616). pDrp1-S616 is the active form of Drp1 that participates in mitochondrial fission. Despite numerous attempts, we saw no pDrp1-S616 on immunofluorescent staining. We suspect pDrp1-S616 is degraded during the tissue harvesting process. Moreover, the increased expression of the Drp1 binding partner MiD51 (Figure 6B) may be sufficient to enhance fission without net increases in total or activated Drp1 expression.

Because we used immunoblotting to detect total cardiac changes in protein expression in whole LV or RV, we cannot determine which cell type(s) within the myocardium (endothelial cells, fibroblasts, vascular smooth muscle cells, cardiac myocytes or inflammatory cells) contribute(s) to the upregulation of the PKM2 to PKM1 ratio.

Finally, because this is meant to be a hypothesis-generating study, we have not attempted to intervene and alter expression of PKM1/PKM2 or MiD51 to determine the effects on group 2 PH or ventricular function. *In vivo* interventions will be required to determine whether these novel mitochondrial arrangements are adaptive or pathologic, which will determine the potential value of these proteins as therapeutic targets in group 2 PH.

## Author Contributions

PX contributed to the design of the study, wrote the first draft of the manuscript, generated the figures, performed rodent banding surgeries, echocardiography, hemodynamic studies, Western blots, and analyzed and interpreted all data. LT contributed to the design of the study, performed tissue harvesting, analyzed and interpreted histological, TMRM, and TEM image data. KD-S contributed to the design of the study, assisted with the generation and analysis of the PDH data. K-HC and AD assisted with running the Western blots. JM performed microscopic analysis of tissue specimens and took the TMRM confocal images. MN-H assisted in the SAB surgery and post-operative care of the animals. AM performed pre-operative equipment preparation, assisted in the SAB surgery, and provided post-operative treatments and animal care. FP assisted in the hemodynamic studies and analysis of the hemodynamic data. SA contributed to the conception and design of the study, oversaw the writing and editing and funded the study. All authors contributed to manuscript revision, read and approved the submitted version.

### Conflict of Interest Statement

The authors declare that the research was conducted in the absence of any commercial or financial relationships that could be construed as a potential conflict of interest.

## Supplementary Material

Supplemental Figure 1 Supra-coronary aortic banding (SAB) group 2 pulmonary hypertension rat model experimental design.

Supplemental Figure 2 Supra-coronary aortic banding surgery. **(A)** Incision made at 3rd intercostal space and held open with three surgical hooks; **(B)** the aorta was located; **(C)** the connective tissue between aorta and pulmonary artery was carefully removed with a pair of blunt tweezers; **(D)** a small metal clip was applied to the isolated aorta, constricting it by 50–60%; **(E)** the clipped aorta and surrounding structure; **(F)** a 16-gauge chest tube was inserted and the chest was closed by 4–0 Vicryl suture; **(G)** closing the outer muscle layer with simple continuous suture; **(H)** closing the outer skin with simple interrupted suture and 2–3 surgical clips.

Supplemental Figure 3 Heart rate and respiratory rate 4 weeks post banding surgery. **(A)** Representative electrocardiogram and respiratory trace of sham and supra-coronary aortic banding (SAB) rat. **(B)** Respiratory rate is significantly increased in SAB vs. sham rats. **(C)** Heart rate is not significantly increased in SAB vs. sham rats.

Supplemental Figure 4 Western blot showing no change in mitochondrial dynamics of 49 kDa protein (MiD49) in the **(A)** left ventricle (LV) and **(B)** right ventricle (RV) of supra-coronary aortic banding (SAB) rats vs. sham rats. Each band represents a unique animal. Two experimental cohorts were done; hence two groups are shown, labeled as 1 and 2. The same vinculin loading control is used for proteins probed on the same membrane.

Supplemental Figure 5 Western blot showing no significant change of phospho-pyruvate dehydrogenase (pPDH) to pyruvate dehydrogenase (PDH) ratio in the **(A)** left ventricle (LV) and **(B)** right ventricle (RV) of supra-coronary aortic banding (SAB) rats vs. sham rats. Each band represents a unique animal. Two experimental cohorts were done; hence two groups are shown, labeled as 1 and 2. The same vinculin loading control is used for proteins probed on the same membrane.

Supplemental Figure 6 Pyruvate dehydrogenase (PDH) enzyme activity dipstick assay showing no significant change in the **(A)** left ventricle (LV) and the **(B)** right ventricle (RV) PDH enzyme activity.
