# Supplementary figures and images for: Biventricular Increases in Mitochondrial Fission Mediator (MiD51) and Proglycolytic Pyruvate Kinase (PKM2) Isoform in Experimental Group 2 Pulmonary Hypertension-Novel Mitochondrial Abnormalities
Source: Front Cardiovasc Med. 2019 Jan 25;5:195. doi: 10.3389/fcvm.2018.00195 (PMC6355690; doi:10.3389/fcvm.2018.00195)

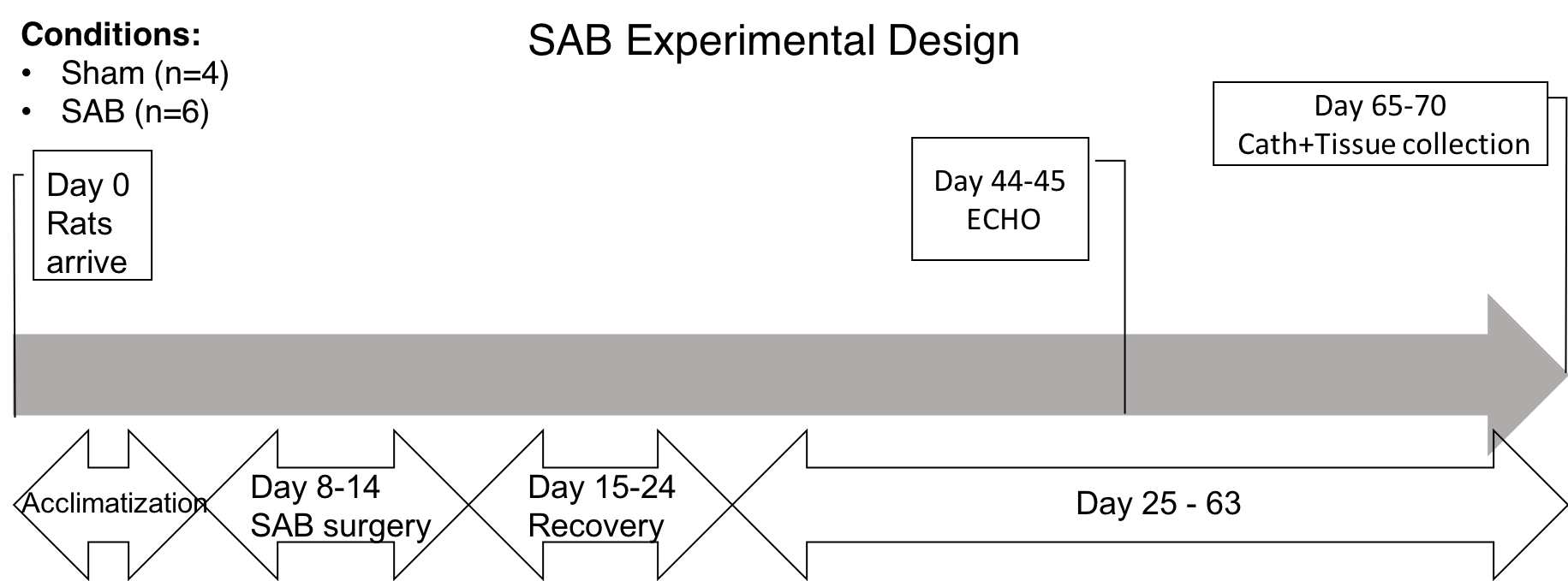

Supplement: Supplemental Figure 1 — Supra-coronary aortic banding (SAB) group 2 pulmonary hypertension rat model experimental design. [file Image_1.TIF]

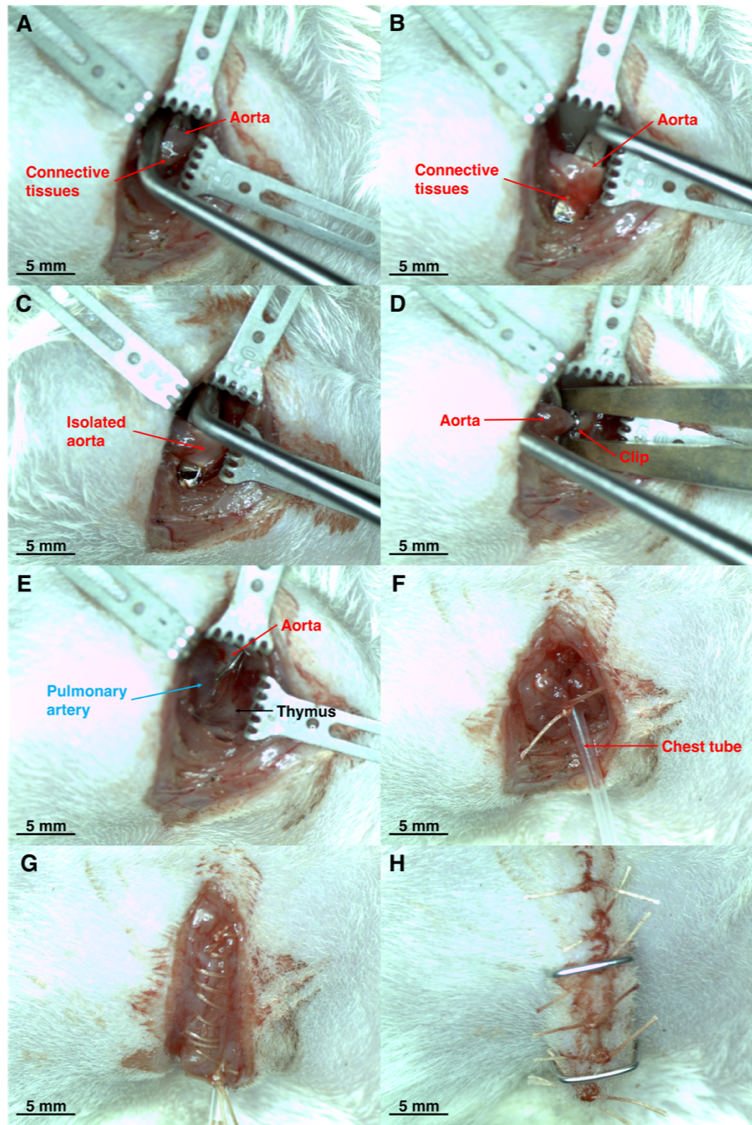

Supplement: Supplemental Figure 2 — Supra-coronary aortic banding surgery. (A) Incision made at 3rd intercostal space and held open with three surgical hooks; (B) the aorta was located; (C) the connective tissue between aorta and pulmonary artery was carefully removed with a pair of blunt tweezers; (D) a small metal clip was applied to the isolated aorta, constricting it by 50–60%; (E) the clipped aorta and surrounding structure; (F) a 16-gauge chest tube was inserted and the chest was closed by 4–0 Vicryl suture; (G) closing the outer muscle layer with simple continuous suture; (H) closing the outer skin with simple interrupted suture and 2–3 surgical clips. [file Image_2.TIF]

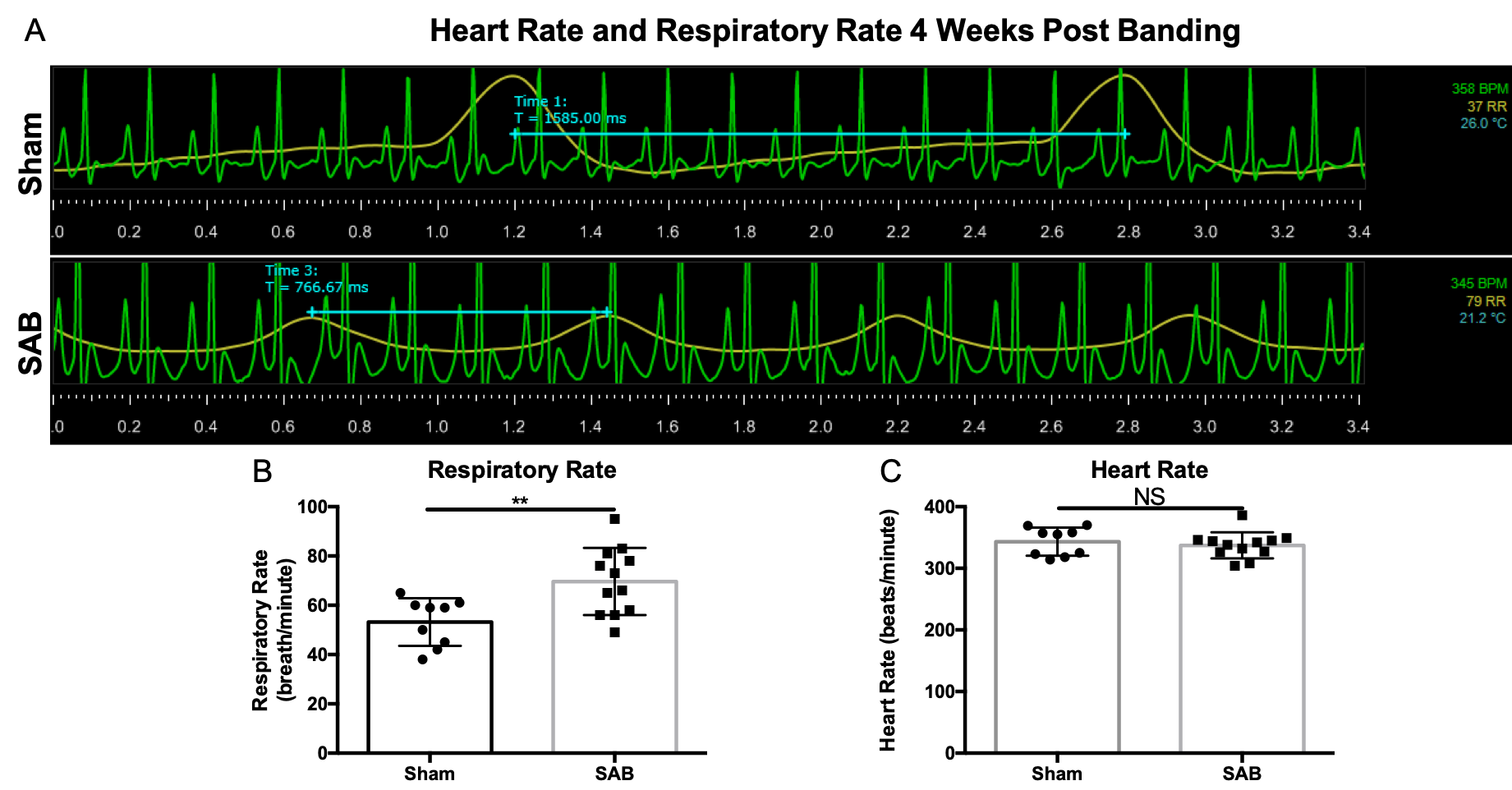

Supplement: Supplemental Figure 3 — Heart rate and respiratory rate 4 weeks post banding surgery. (A) Representative electrocardiogram and respiratory trace of sham and supra-coronary aortic banding (SAB) rat. (B) Respiratory rate is significantly increased in SAB vs. sham rats. (C) Heart rate is not significantly increased in SAB vs. sham rats. [file Image_3.TIF]

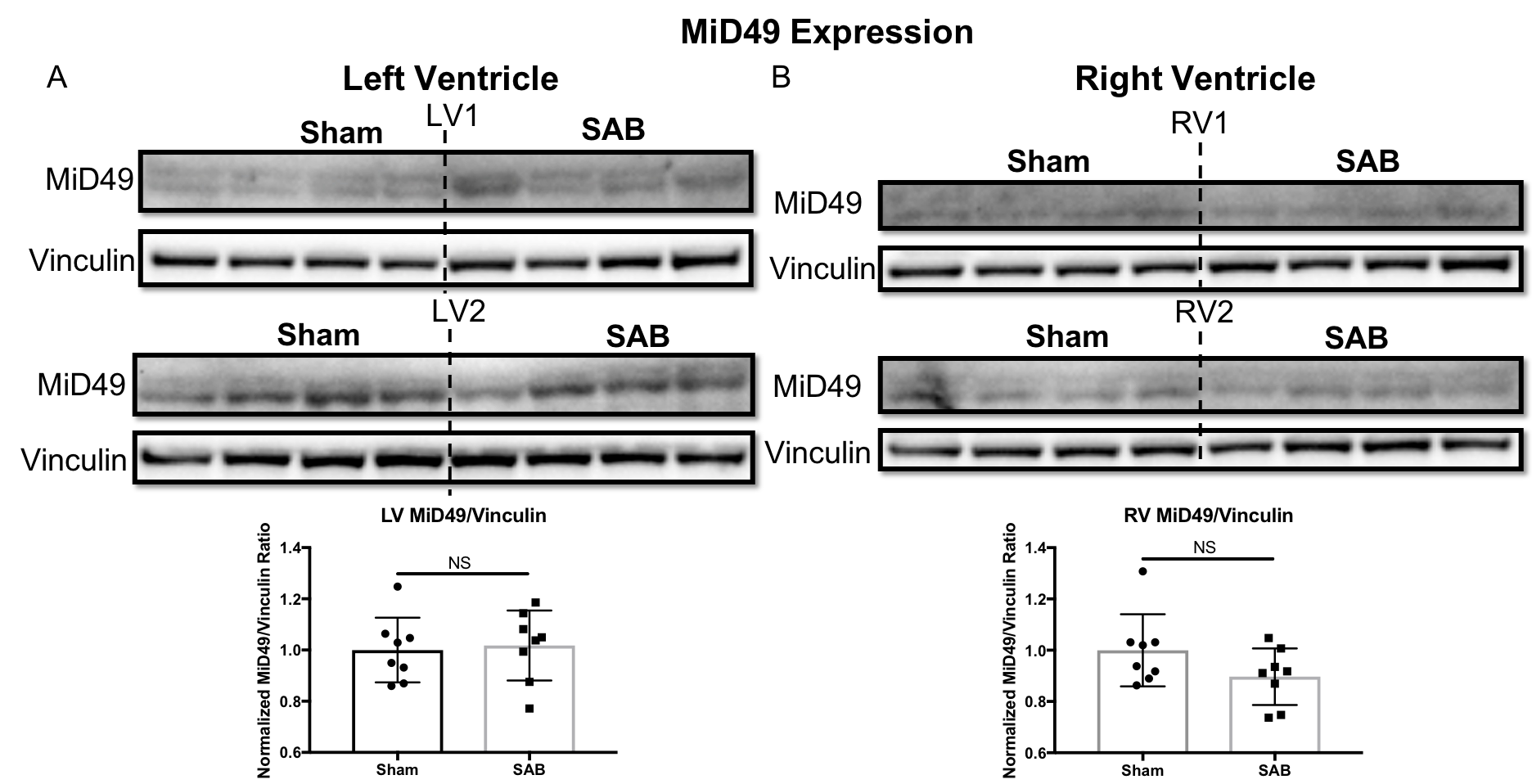

Supplement: Supplemental Figure 4 — Western blot showing no change in mitochondrial dynamics of 49 kDa protein (MiD49) in the (A) left ventricle (LV) and (B) right ventricle (RV) of supra-coronary aortic banding (SAB) rats vs. sham rats. Each band represents a unique animal. Two experimental cohorts were done; hence two groups are shown, labeled as 1 and 2. The same vinculin loading control is used for proteins probed on the same membrane. [file Image_4.TIF]

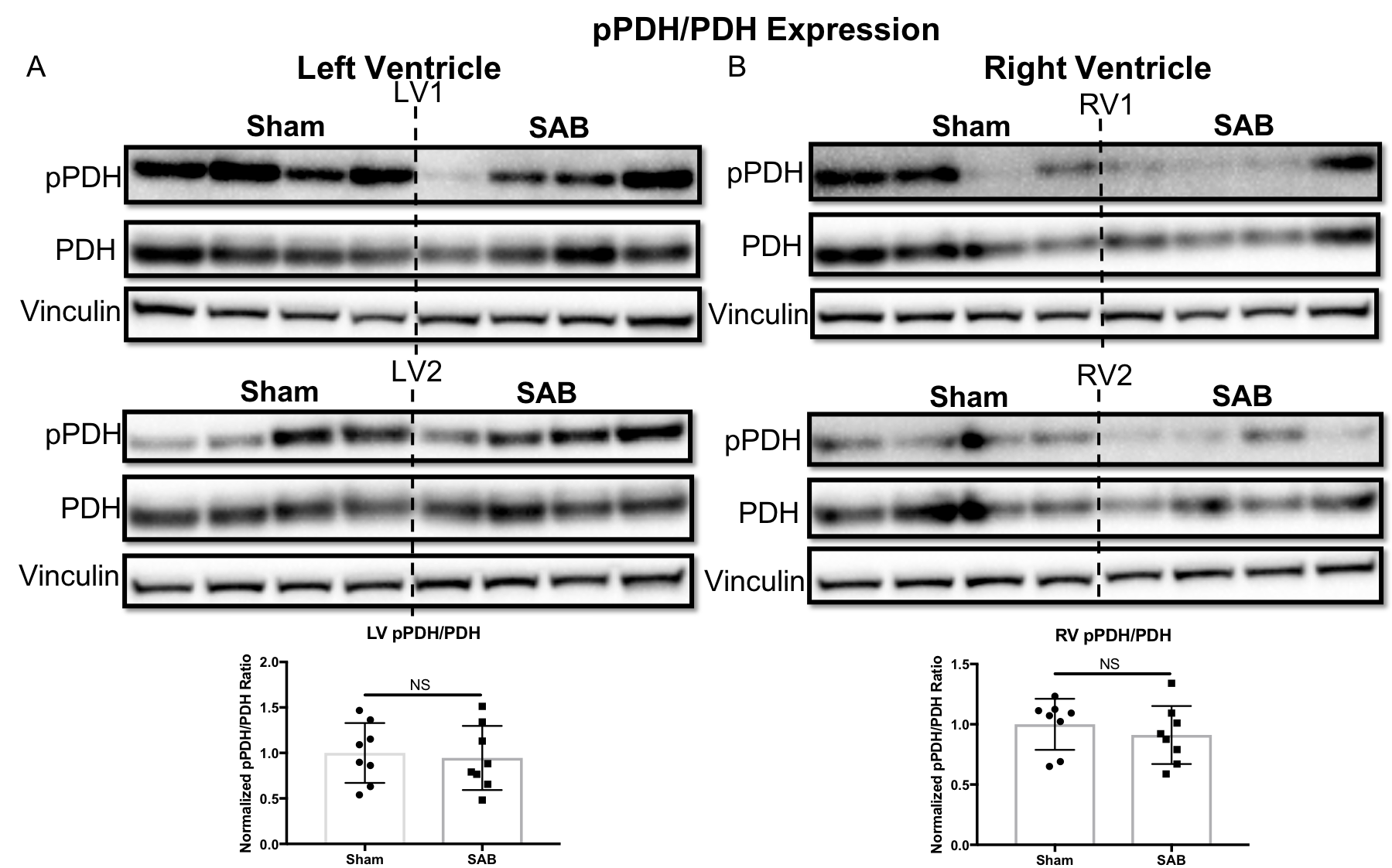

Supplement: Supplemental Figure 5 — Western blot showing no significant change of phospho-pyruvate dehydrogenase (pPDH) to pyruvate dehydrogenase (PDH) ratio in the (A) left ventricle (LV) and (B) right ventricle (RV) of supra-coronary aortic banding (SAB) rats vs. sham rats. Each band represents a unique animal. Two experimental cohorts were done; hence two groups are shown, labeled as 1 and 2. The same vinculin loading control is used for proteins probed on the same membrane. [file Image_5.TIF]

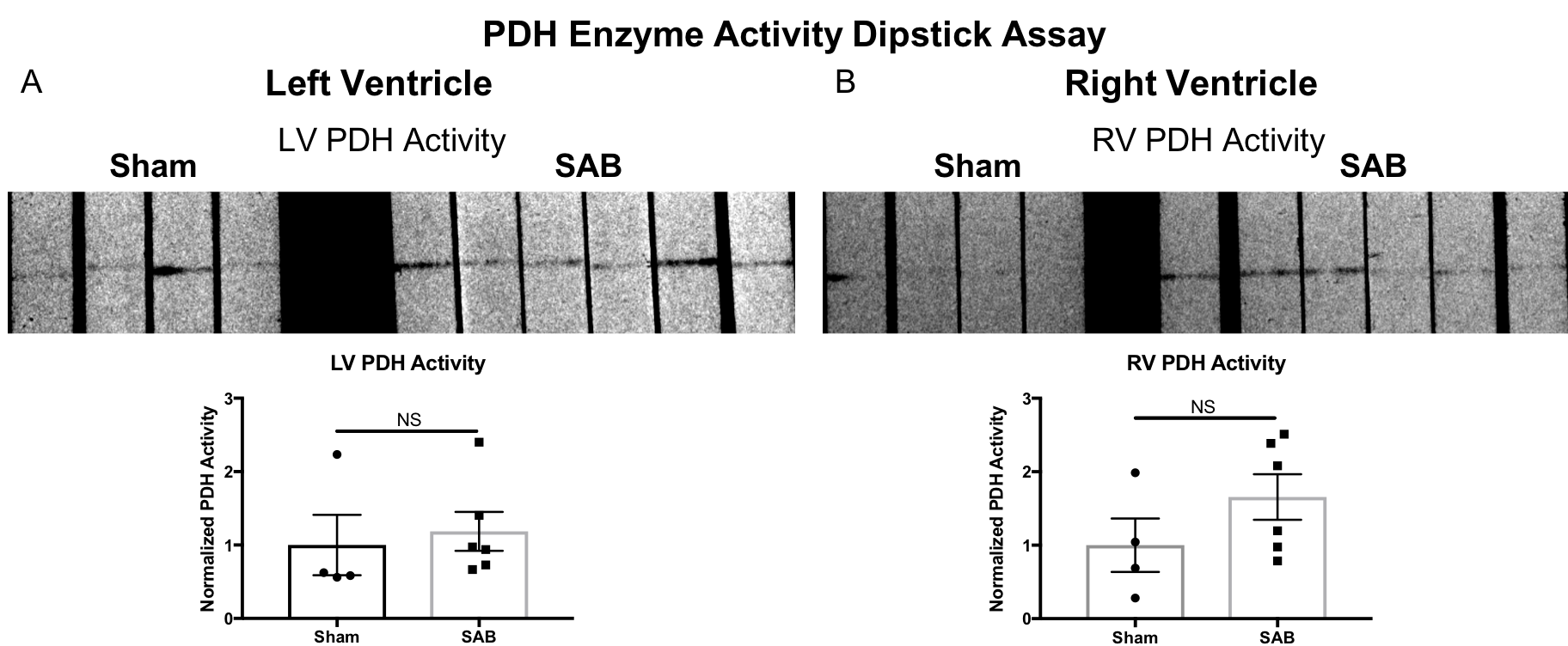

Supplement: Supplemental Figure 6 — Pyruvate dehydrogenase (PDH) enzyme activity dipstick assay showing no significant change in the (A) left ventricle (LV) and the (B) right ventricle (RV) PDH enzyme activity. [file Image_6.TIF]
